# Comparison of the efficacy and safety of 755-nm picosecond alexandrite laser with diffractive lens array vs. fractional microneedle radiofrequency for the treatment of facial photoaging: a retrospective study

**DOI:** 10.3389/fmed.2026.1913396

**Published:** 2026-08-31

**Authors:** Xixin Wu, Houhuang Qiu, Jingchun Chen, Yunkai Zhu, Jialu Xu, Xi Chen, Yutong Wu, Fangfang Wang, Ping Zhong, Qiusheng Lin, Fei Li, John Chen, Hongduo Chen, Tianhua Xu

**Affiliations:** 1 Medical Cosmetic Center, Shenzhen Nanshan People's Hospital/Affiliated Nanshan Hospital of Shenzhen University, Shenzhen, Guangdong, China; 2 HealthySkin/Forefront Dermatology, Tucson, AZ, United States; 3 Department of Dermatology, The First Hospital of China Medical University, Shenyang, China; 4 Key Laboratory of Immunodermatology, Ministry of Education, and National Health Commission, National Joint Engineering Research Center for Theranostics of Immunological Skin Diseases, Shenyang, China

**Keywords:** DLA, facial photoaging, MNRF, PsaL, VISIA

## Abstract

**Objective:**

To compare the efficacy and safety of a 755-nm picosecond alexandrite laser combined with a diffractive lens array (PSAL-DLA) vs. fractional microneedle radiofrequency (MNRF) in the treatment of facial photoaging.

**Methods:**

This retrospective study enrolled 51 patients with facial photoaging (Glogau scale ≥ II), including 22 in the PSAL-DLA group and 29 in the MNRF group. Objective assessments were performed at baseline and within 3 months post-treatment using the VISIA skin analysis system (spots, wrinkles, texture, pores, UV spots, brown spots, red areas, porphyrins). Subjective evaluation was conducted independently by two board-certified physicians using the Modified Fitzpatrick Wrinkle Scale (MFWS) and Global Aesthetic Improvement Scale (GAIS). Analysis of covariance (ANCOVA) was used to compare between-group efficacy, with baseline VISIA scores, age, sex, skin type, and actual follow-up duration as covariates. Treatment-related adverse events were also recorded.

**Results:**

The PSAL-DLA group demonstrated significant improvements in brown spots and red areas, and the MNRF group showed a significant reduction in pore scores; however, the between-group differences were not statistically significant (*P* > 0.05). Notably, the MNRF group exhibited a statistically significant worsening of spots from baseline. ANCOVA revealed that the PSAL-DLA group achieved significantly greater improvements in spots total scores (adjusted means: 82.2 vs. 89.3, *P* = 0.004) and UV spots (55.4 vs. 59.6, *P* = 0.022) compared with the MNRF group. Wrinkle improvement correlated positively with baseline severity in both groups. The MNRF group demonstrated a significant within-group improvement in MFWS score, but the between-group comparison with PSAL-DLA showed no significant difference (*P* = 0.103). In the GAIS assessment, 68.18% and 72.41% of subjects in the PSAL-DLA and MNRF groups, respectively, were rated as improved or much improved, with no significant between-group difference (P = 0.779). Subgroup analysis revealed no significant interaction between treatment modality and age or skin type (*P* > 0.05). Adverse reactions were mainly transient erythema and swelling, with no severe hyperpigmentation or scarring observed.

**Conclusion:**

Both PSAL-DLA and MNRF are safe and effective for treating facial photoaging. PSAL-DLA appears superior for pigmented indicators (spots, UV spots), while MNRF also demonstrates efficacy for pores and wrinkles, although between-group differences were not significant. Treatment selection should be individualized based on the patient's predominant photoaging manifestations.

## 1 Introduction

Skin aging encompasses intrinsic chronological aging, driven by genetic and metabolic factors, and extrinsic photoaging, predominantly caused by chronic ultraviolet (UV) radiation exposure. Facial photoaging is characterized by wrinkles, spots, enlarged pores, irregular texture, and skin laxity (1). Histologically, aged skin is typically characterized by impaired skin barrier function, epidermal atrophy, and a reduced number of dermal fibroblasts (2). UV radiation triggers DNA damage, oxidative stress, and inflammatory responses in skin cells (3). The generation of reactive oxygen species (ROS) and the activation of specific signaling cascades promote the degradation of the extracellular matrix, leading to skin aging (4). Studies have shown that compared with healthy controls, patients with facial photoaging exhibit increased facial skin pH and sebum levels, along with decreased elasticity, hydration, and tension (5).

Various therapeutic modalities have been developed for the rejuvenation of photoaged facial skin, primarily focusing on mechanisms such as epidermal repair and stimulation of dermal collagen regeneration (6). These include chemical peels, intense pulsed light, fractional lasers, radiofrequency therapy, and mesotherapy (7–11). Fractional microneedle radiofrequency (MNRF) uses microneedles to puncture the epidermis and precisely deliver radiofrequency energy to the dermis, stimulating the proliferation and remodeling of dermal collagen and elastin through both mechanical and thermal effects (12). The fractional mode of MNRF employs a fractional array of microneedles, generating energy that is evenly distributed in a planar manner, thereby increasing the efficiency of collagen regeneration stimulation (13). It is widely used in clinical practice to improve acne scars, enlarged pores, wrinkles, and skin laxity (14–16). However, the invasiveness of MNRF and the associated post-treatment downtime are undesirable. Consequently, in recent years, there has been a pursuit of minimally invasive or even non-invasive modalities with shorter downtime for the treatment of facial photoaging.

Picosecond lasers are devices capable of emitting laser beams with ultra-short pulse widths on the picosecond order and ultra-high peak power density (17). Acting on target chromophores, they produce both photothermal and photomechanical effects and are commonly used in aesthetic dermatology to treat pigmented lesions and tattoos (18, 19). The 755-nm picosecond alexandrite laser combined with a diffractive lens array (PSAL-DLA) generates a fractional laser energy pattern, inducing laser-induced optical breakdown (LIOB) deep in the epidermis, which can promote skin regeneration and collagen remodeling (20). A prospective study demonstrated that PSAL-DLA effectively improved irregular facial skin texture and hyperpigmentation (21). Other studies have reported that PSAL-DLA is also effective for treating facial wrinkles and enlarged pores (22).

Based on the above findings, PSAL-DLA can be applied to the treatment of facial photoaging. Moreover, PSAL-DLA is associated with tolerable pain, a virtually non-invasive epidermis, and a short recovery period. The primary objective of this retrospective study was to compare the efficacy and safety of PSAL-DLA vs. MNRF for the treatment of facial photoaging, aiming to provide a new perspective with less trauma and shorter downtime for facial photoaging treatment.

## 2 Materials and methods

### 2.1 Patient information

This was a retrospective study that enrolled patients who underwent a single treatment session with either PSAL-DLA or MNRF for facial photoaging at the Department of Medical Aesthetics, Shenzhen Nanshan People's Hospital, between January 2024 and April 2026. Of the 98 patients who met the inclusion criteria for the 755-nm picosecond alexandrite laser with diffractive lens array treatment, 76 were excluded: 51 had received other photoaging-related treatments during the follow-up period, and 25 were lost to follow-up, leaving 22 patients in the final analysis. Of the 133 patients who met the inclusion criteria for the fractional microneedle radiofrequency treatment, 104 were excluded: 80 had received other photoaging-related treatments during the follow-up period, and 24 were lost to follow-up, leaving 29 patients in the final analysis. This retrospective study adhered to the principles of the Declaration of Helsinki and was approved by the Ethics Committee of Shenzhen Nanshan People's Hospital, which authorized the use of de-identified clinical data. Written informed consent was obtained from all participants prior to treatment in this trial.

### 2.2 Inclusion and exclusion criteria

#### 2.2.1 Inclusion criteria

Patients evaluated by two independent dermatologists using the Glogau Photoaging Scale with a score ≥ II (23); age 18–60 years; patients with complete clinical and photographic records at baseline available for retrospective evaluation.

#### 2.2.2 Exclusion criteria

Pregnant or lactating women; patients with hepatic or renal insufficiency, bone marrow suppression, mental disorders, or other severe systemic diseases; patients with a history of keloid scarring or active infectious skin disease on the face; patients who had used photosensitizing agents, including retinoids, within the past 6 months; patients who had received other facial photoaging-related treatments within the past 6 months or during the study period.

### 2.3 Treatment protocol

Prior to treatment, a compound topical anesthetic cream containing 2.5% lidocaine and 2.5% prilocaine (Tongfang Pharmaceutical Group Co., Ltd., Beijing, China) was applied to the treatment area for 40–50 min. Treatment was performed after cleansing and disinfection.

In the picosecond laser treatment group, the first step involved a flat scanning mode using the PSAL (Picosure pro, Cynosure, Westford, MA, USA) with a fluence range of 0.4–0.6 J/cm^2^, a pulse duration of 750 ps, a spot size of 8–10 mm, and a frequency of 10 Hz. The treatment area was scanned once, with a total of approximately 1,000–1,500 pulses delivered. The second step was performed immediately after the first step, using the PSAL combined with DLA (Focus, Cynosure, Westford, MA, USA) with a fluence range of 1.3–1.5 J/cm^2^, a pulse duration of 750 ps, a spot size of 5 mm, and a frequency of 5 Hz. The treatment area was scanned once, with a total of approximately 800–1,000 pulses delivered. In the fractional microneedle radiofrequency group, all patients were treated with insulated microneedles (Shenzhen Peninsula Medical Co., Ltd., Shenzhen, China). The treatment depth was adjusted according to the skin thickness of different facial regions: 0.8–1.2 mm for the forehead and periorbital area, 1.2–1.4 mm for the zygomatic area, and 1.4–2.0 mm for the cheeks and mandibular area. The energy range was 0.8–1.6 J/cm^2^, the pulse duration range was 120–160 ms, and a total of 800 pulses were delivered to the full face. The treatment endpoint was defined as the appearance of mild to moderate erythema in the treated area.

All patients received standardized post-procedure instructions, including a 15–30 min wet compress after treatment. During the recovery period, patients were advised to maintain adequate moisturization and use a broad-spectrum sunscreen with SPF 50, PA+++, or practice strict sun protection.

### 2.4 Assessment

#### 2.4.1 Objective assessment

Standardized 3D digital imaging was performed using the VISIA^®^ Skin Analysis System (Canfield Scientific, New Jersey, USA) at baseline and at the follow-up visit within 3 months post-treatment (24). The system objectively measures eight absolute parameters of facial skin: spots, wrinkles, texture, pores, UV spots, brown spots, red areas, and porphyrins. The total VISIA score was calculated as the sum of the scores from the frontal, left lateral, and right lateral views.

#### 2.4.2 Subjective assessment

Photographic documentation was performed at baseline and at the post-treatment follow-up visit using the same camera, lighting setup, and patient positioning. All digital photographs were taken with an EOS 850D SLR camera (Canon, Tokyo, Japan). Comparison of pre- and post-treatment photographs was conducted using the Modified Fitzpatrick Wrinkle Scale (MFWS) (25) and Global Aesthetic Improvement Scale (GAIS) for comprehensive efficacy evaluation. Evaluations were performed independently by two board-certified aesthetic physicians who were blinded to the treatment allocation. (Global Aesthetic Improvement Scale: 0 = worsening, 1 = no change, 2 = improved, 3 = markedly improved, 4 = dramatically improved).

#### 2.4.3 Safety assessment

Adverse skin reactions following each treatment session, including erythema, edema, burning sensation, stinging, post-inflammatory hyperpigmentation, or hypopigmentation, were observed and recorded. The same physician examined patients at the follow-up visits.

### 2.5 Statistical methods

Data were analyzed using SPSS 27.0 software (Version 27.0; IBM Corp., Armonk, NY, USA). Normality of continuous variables was assessed using the Shapiro-Wilk test. Normally distributed continuous variables are presented as mean ± standard deviation (SD), while non-normally distributed continuous variables are presented as median (P25, P75). Baseline characteristics were compared between groups using the independent samples t-test for normally distributed continuous variables, the Mann-Whitney U test for non-normally distributed continuous variables, and the chi-square test or Fisher's exact test for categorical variables. For efficacy analysis, analysis of covariance (ANCOVA) was performed with the post-treatment VISIA total score as the dependent variable, treatment modality as a fixed factor, and baseline VISIA total score, age, sex, Fitzpatrick skin type, and actual follow-up weeks as covariates, to adjust for baseline imbalances and differences in follow-up time. Within-group changes from baseline were explored using the Wilcoxon signed-rank test. The GAIS responder rates were compared using the chi-square test or Fisher's exact test. Linear regression analysis was employed to examine the relationship between baseline total VISIA scores and post-treatment improvement values. A two-sided p < 0.05 was considered statistically significant.

## 3 Results

### 3.1 General characteristics of patients

**Figure 1:**
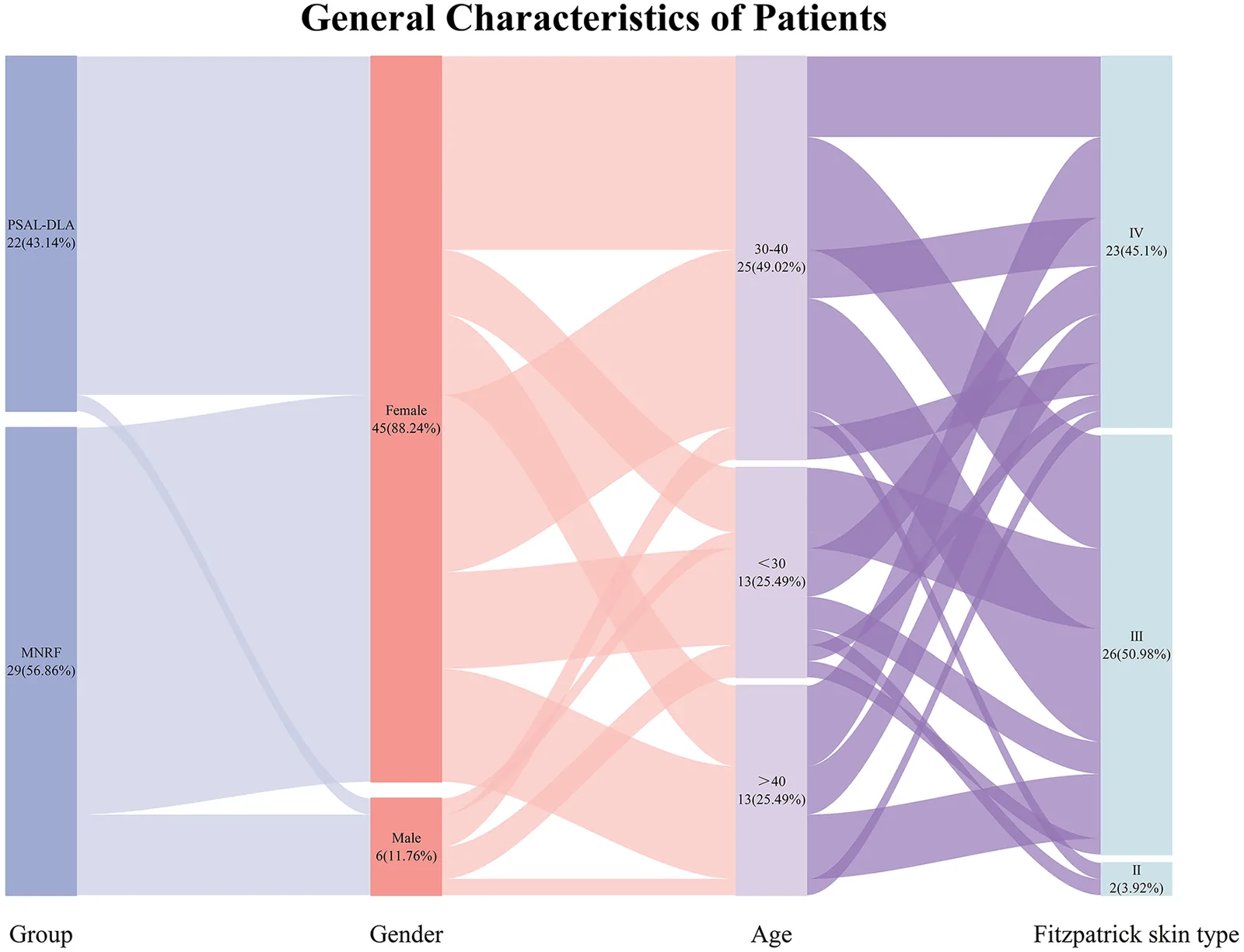
Baseline demographic and clinical characteristics of the study population.

A total of 51 patients were enrolled in this study, 22 (43.14%) were assigned to the PSAL-DLA group, while the remaining 29 (56.86%) were in the MNRF group. The cohort showed a significant female predominance, consisting of 45 females (88.24%) and 6 males (11.76%). The majority of patients were aged between 30 and 40 years (25 patients, 49.02%). Patients under 30 years and over 40 years accounted for 13 cases (25.49%) each. Most patients had Fitzpatrick skin type III or IV, 26 patients (50.98%) had type III skin, 23 (45.1%) had type IV skin, and only 2 cases (3.92%) presented with type II skin (Figure 1).

Baseline characteristics are summarized in Table 1. The two groups were comparable in terms of age, gender, Fitzpatrick skin type, and most VISIA sub-scores (wrinkles, texture, pores, brown spots, red areas, porphyrins), as well as follow-up duration (all *P* > 0.05). However, a borderline between-group difference was noted for the baseline VISIA spots score (*P* = 0.060), and a statistically significant baseline imbalance was observed for the UV spots score (*P* = 0.030). These imbalances were subsequently adjusted for in the ANCOVA models by including baseline scores as covariates.

**Table 1:** Comparative analysis of Baseline characteristics.

Characteristic	PSAL-DLA (*n* = 22)	MNRF (*n* = 29)	Statistical Test	*P*-Value
**Age (years)**	35.500 ± 7.806	36.862 ± 9.764	t = −0.537	0.594
Gender (*n*, %)			Fisher	0.218
Male	1 (4.55%)	5 (17.24%)		
Female	21 (95.45%)	24 (82.76%)		
Fitzpatrick skin type (*n*, %)			χ^2^ = 0.002	0.964
Type II-III	12 (54.55%)	16 (55.17%)		
Type IV	10 (45.45%)	13 (44.83%)		
VISIA Total score at baseline				
Spots	80.897 (68.559, 89.743)	88.612 (77.867, 100.550)	Z = −1.883	0.060
Wrinkles	52.749 (43.105, 75.785)	59.284 (45.257, 71.675)	Z = −0.494	0.621
Texture	21.376 (12.478, 30.306)	26.919 (16.913, 35.011)	Z = −1.122	0.262
Pores	76.811 (54.740, 116.880)	72.264 (52.458, 121.634)	Z = −0.038	0.970
UV spots	51.166 (45.205, 56.813)	58.034 (49.882, 70.513)	Z = −2.168	0.030
Brown spots	49.860 (44.029, 57.396)	53.072 (46.371, 67.754)	Z = −1.103	0.270
Red areas	31.662 (27.787, 34.393)	32.032 (30.026, 37.217)	Z = −0.761	0.447
Porphyrins	53.306 (21.931, 75.449)	50.095 (22.197, 82.594)	Z = −0.304	0.761
**Follow-up time (week)**	8 (7, 12)	8 (4, 10)	Z = −0.956	0.339

### 3.2 Primary outcomes

**Figure 2:**
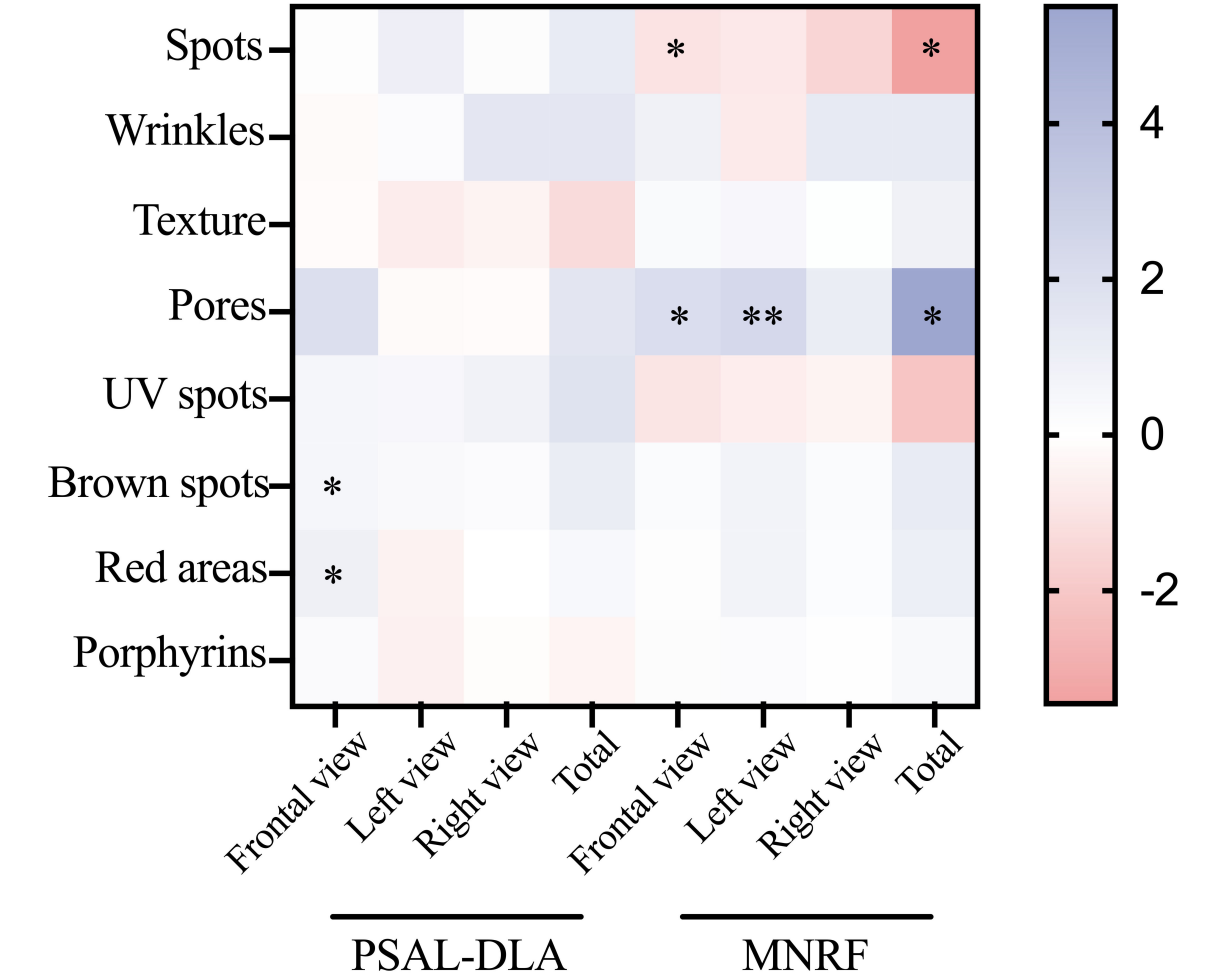
Within-group changes in VISIA absolute scores from baseline to post-treatment in the PSAL-DLA and MNRF groups. The heatmap displays the changes in scores for various skin parameters (Spots, Wrinkles, Texture, Pores, UV spots, Brown spots, Red areas, and Porphyrins) across different facial views. The color gradient represents the magnitude of change (red indicates a deterioration, blue indicates an improvement). ^*^ means *p* < 0.05, ^**^ means *p* < 0.01.

Figure 2 illustrates the within-group changes in absolute scores of various VISIA skin parameters before and after treatment in the PSAL-DLA and MNRF groups. The heatmap colors correspond to the difference between the baseline score and the post-treatment score: blue areas represent a positive difference (improvement), and red areas represent a negative difference (worsening). In the PSAL-DLA group, brown spots and red areas showed significant improvement on the frontal view (Z = −2.224, *p* = 0.026; Z = −2.321, *p* = 0.020). In the MNRF group, pores demonstrated significant improvement on the frontal view, left lateral view, and in the total score (Z = −2.000, *p* = 0.045; Z = −2.930, *p* = 0.003; Z = −2.130, *p* = 0.033). However, the MNRF group showed a statistically significant increase in spots on the frontal view and total score from baseline (Z = −2.260, *P* = 0.024; Z = −2.195, *P* = 0.028), indicating a statistically significant worsening.

**Figure 3:**
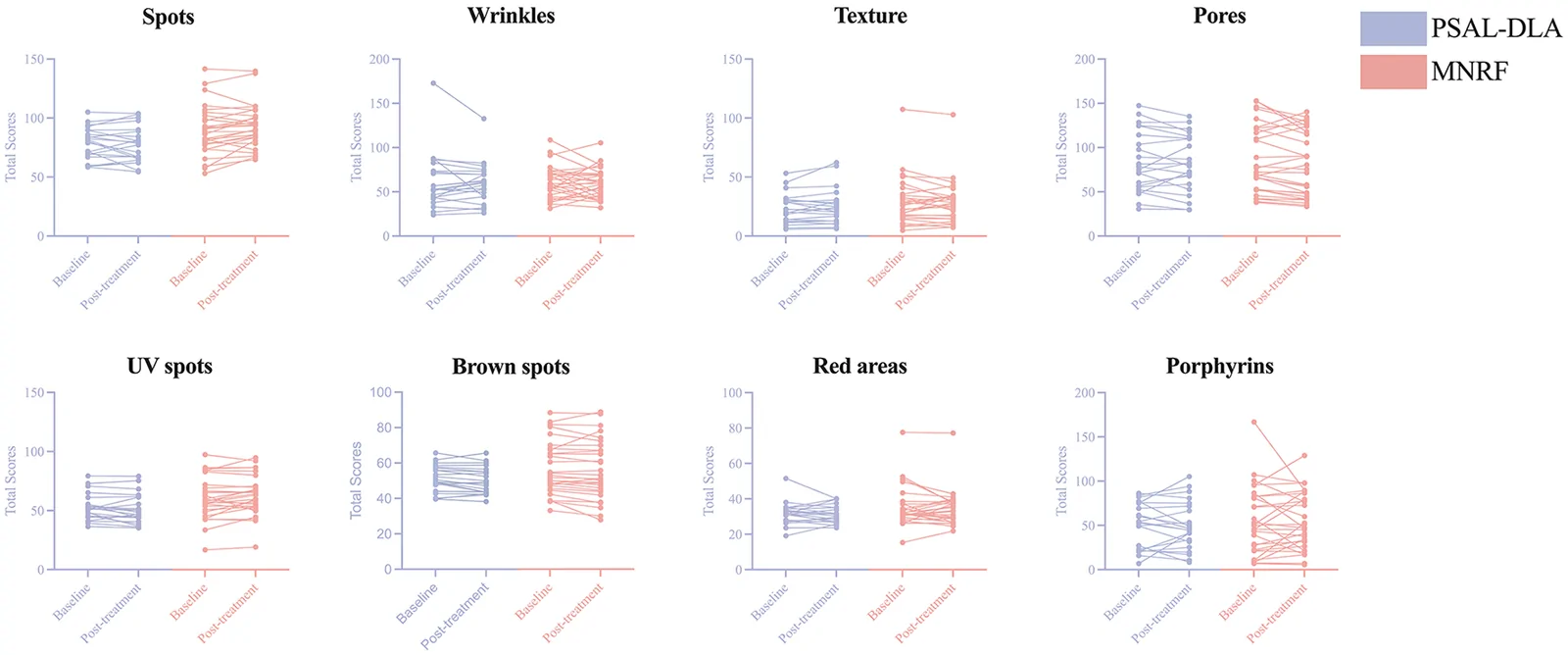
Individual paired changes in VISIA total scores after PSAL-DLA and MNRF treatment. Each panel displays paired data points for individual patients before (Baseline) and after (Post-treatment) treatment for the indicated skin parameters. Blue lines indicate the PSAL-DLA group, and red lines indicate the MNRF group.

In individual paired change trajectories of the total scores for each VISIA parameter before and after treatment in the PSAL-DLA and MNRF groups. An upward sloping line indicates an increase in the total score for that parameter (worsening), while a downward sloping line indicates a decrease in the total score (improvement). In the MNRF group, the change trajectories for spots were predominantly upward sloping for most individuals, while those for pores were predominantly downward sloping, consistent with the within-group comparisons described above (Figure 3).

**Figure 4:**
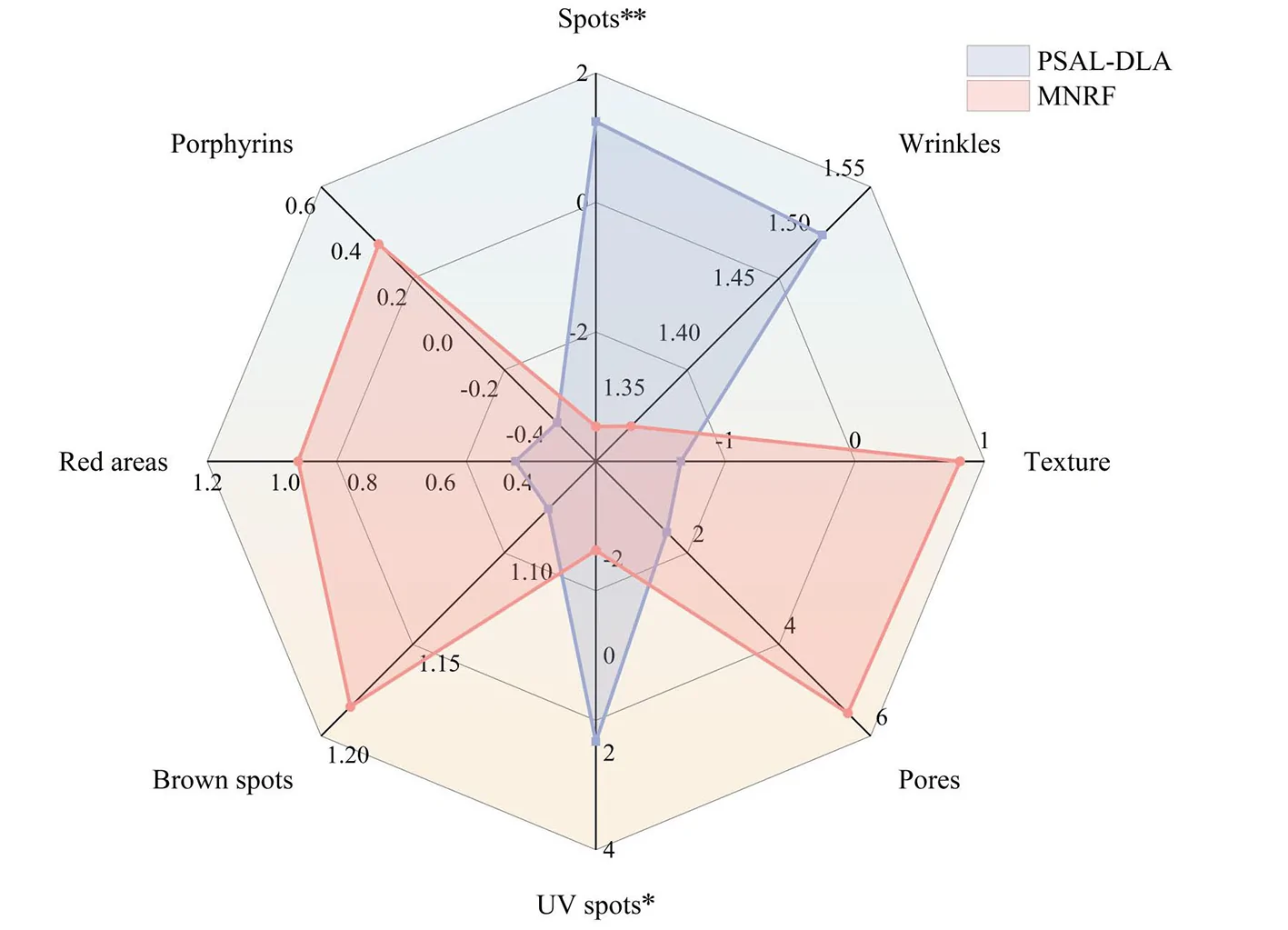
Comparison of VISIA total score improvements between the PSAL-DLA and MNRF groups. The radar chart visualizes the mean improvement scores across eight skin parameters. Blue areas represent the PSAL-DLA group, and red areas represent the MNRF group. ^*^ means *p* < 0.05, ^**^ means *p* < 0.01.

Figure 4 uses a radar chart to visually present the average degree of improvement in each VISIA parameter for the PSAL-DLA and MNRF groups (improvement = baseline score – post-treatment score). The outward extension of the graph represents the magnitude of improvement. After adjusting for baseline total VISIA scores and confounding factors, the adjusted marginal mean of the post-treatment spots total score was 82.198 (95% CI: 78.820–85.577) in the PSAL-DLA group and 89.257 (95% CI: 86.352–92.161) in the MNRF group. The improvement in spots was significantly greater in the PSAL-DLA group than in the MNRF group (F = 9.324, *P* = 0.004, partial η^2^ = 0.175). The adjusted marginal mean of the post-treatment UV spots total score was 55.429 (95% CI: 52.880–57.979) in the PSAL-DLA group and 59.560 (95% CI: 57.367–61.753) in the MNRF group. The improvement in UV spots was significantly greater in the PSAL-DLA group than in the MNRF group (F = 5.619, *P* = 0.022, partial η^2^ = 0.113).

**Figure 5:**
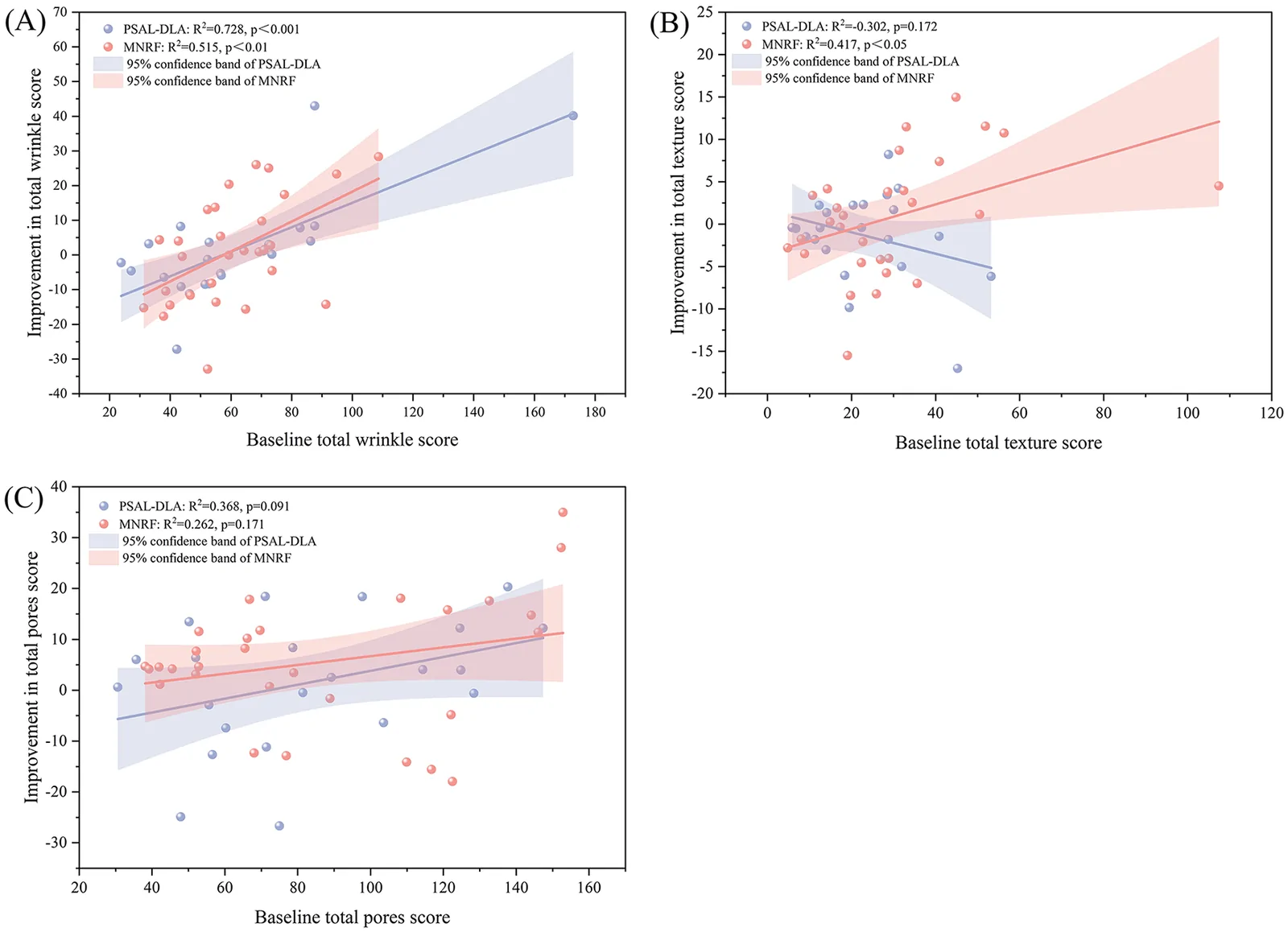
Association between baseline VISIA total scores and treatment-induced improvements in the PSAL-DLA and MNRF groups. Panels **(A–C)** depict the linear regression analyses for Wrinkles, Texture, and Pores, respectively. Scatter plots show individual patient data points (blue dots for PSAL-DLA, red dots for MNRF). Solid lines represent the linear regression fit, and shaded regions indicate the 95% confidence bands.

Figure 5 illustrates the correlation between the baseline total VISIA score (baseline severity) for different parameters and the degree of improvement after treatment. For wrinkles, both treatment modalities showed a significant positive correlation (R^2^ = 0.728, *p* < 0.001; R^2^ = 0.515, *p* < 0.01). For texture, the MNRF group showed a significant positive correlation (R^2^ = 0.417, *p* < 0.05). For pores, no significant linear correlation was observed in either group (*p* > 0.05).

Subgroup analysis revealed no significant interaction between treatment modality and age or Fitzpatrick skin type (*P* for interaction > 0.05), suggesting that the differences in efficacy for wrinkles, texture, and pores between the two treatments did not vary significantly by age or skin type. Numerically, subjects aged < 40 years and those with Fitzpatrick skin type II-III in the MNRF group showed a greater magnitude of improvement in the total pore score, but this difference did not reach statistical significance compared with the PSAL-DLA group (*P* > 0.05). However, given the limited sample size of each subgroup, statistical power may have been insufficient to detect clinically meaningful subgroup differences. Therefore, these results should be interpreted as exploratory, and the possibility of differential treatment effects across subgroups cannot be excluded (Tables 2–4).

**Table 2:** Subgroup analysis based on VISIA total wrinkle scores [M (P25, P75)].

Subgroup	Number (*n*)	VISIA total wrinkle scores improvements		95% confidence interval	*P*-Value	*p* value for interaction
		PSAL-DLA	MNRF			
Age (years)						
< 30	13	−2.210 (-16.790, 3.425)	0.447 (-11.335, 10.829)	(-22.328, 10.288)	0.558	0.606
30–40	25	−0.043 (-7.552, 8.152)	−0.421 (-14.435, 9.572)	(-8.138, 17.058)	0.663	
>40	13	0.187 (-7.477, 22.114)	5.648 (-10.373, 24.624)	(-26.557, 23.483)	0.770	
Fitzpatrick skin type						
Type II-III	28	−0.043 (-5.967, 3.528)	2.014 (-10.766, 12.260)	(-12.352, 10.784)	0.642	0.812
Type IV	23	−2.546 (-8.507, 7.971)	0.914 (-14.864, 21.856)	(-13.507, 16.181)	0.804	

**Table 3:** Subgroup analysis based on VISIA total texture scores [M (P25, P75)].

Subgroup	Number (*n*)	VISIA total texture scores improvements		95% confidence interval	*P*-Value	*p* value for interaction
		PSAL-DLA	MNRF			
Age (years)						
< 30	13	−0.415 (-5.175, 0.485)	3.598 (-2.546, 9.113)	(-11.730, 0.982)	0.079	0.200
30-40	25	0.632 (-2.706, 2.299)	−1.736 (-5.146, 2.486)	(-4.134, 6.298)	0.446	
>40	13	−1.802 (-11.588, 1.021)	1.846 (-5.757, 6.679)	(-15.102, 4.214)	0.306	
Fitzpatrick skin type						
Type II-III	28	−0.464 (-2.706, 1.616)	−0.735 (-4.006, 4.120)	(-5.758, 3.744)	0.816	0.486
Type IV	23	−0.934 (-5.288, 2.607)	1.919 (-4.287, 5.959)	(-9.050, 1.940)	0.239	

**Table 4:** Subgroup analysis based on VISIA total pores scores [M (P25, P75)].

Subgroup	Number (*n*)	VISIA total pores scores improvements		95% confidence interval	*P*-Value	*p* value for interaction
		PSAL-DLA	MNRF			
Age (years)						
< 30	13	3.957 (-13.884, 15.982)	11.658 (-7.318, 16.845)	(-29.007, 14.659)	0.558	0.521
30–40	25	0.079 (-10.237, 11.245)	4.572 (-2.026, 13.612)	(-15.454, 4.390)	0.301	
>40	13	6.025 (1.740, 12.410)	3.657 (-0.922, 4.683)	(-8.370, 15.520)	0.306	
Fitzpatrick skin type						
Type II–III	28	1.053 (-12.289, 12.209)	6.464 (3.622, 14.027)	(-18.976, 1.808)	0.126	0.160
Type IV	23	5.048 (-2.038, 12.214)	0.747 (-13.221, 14.155)	(-9.524, 12.856)	0.577	

**Figure 6:**
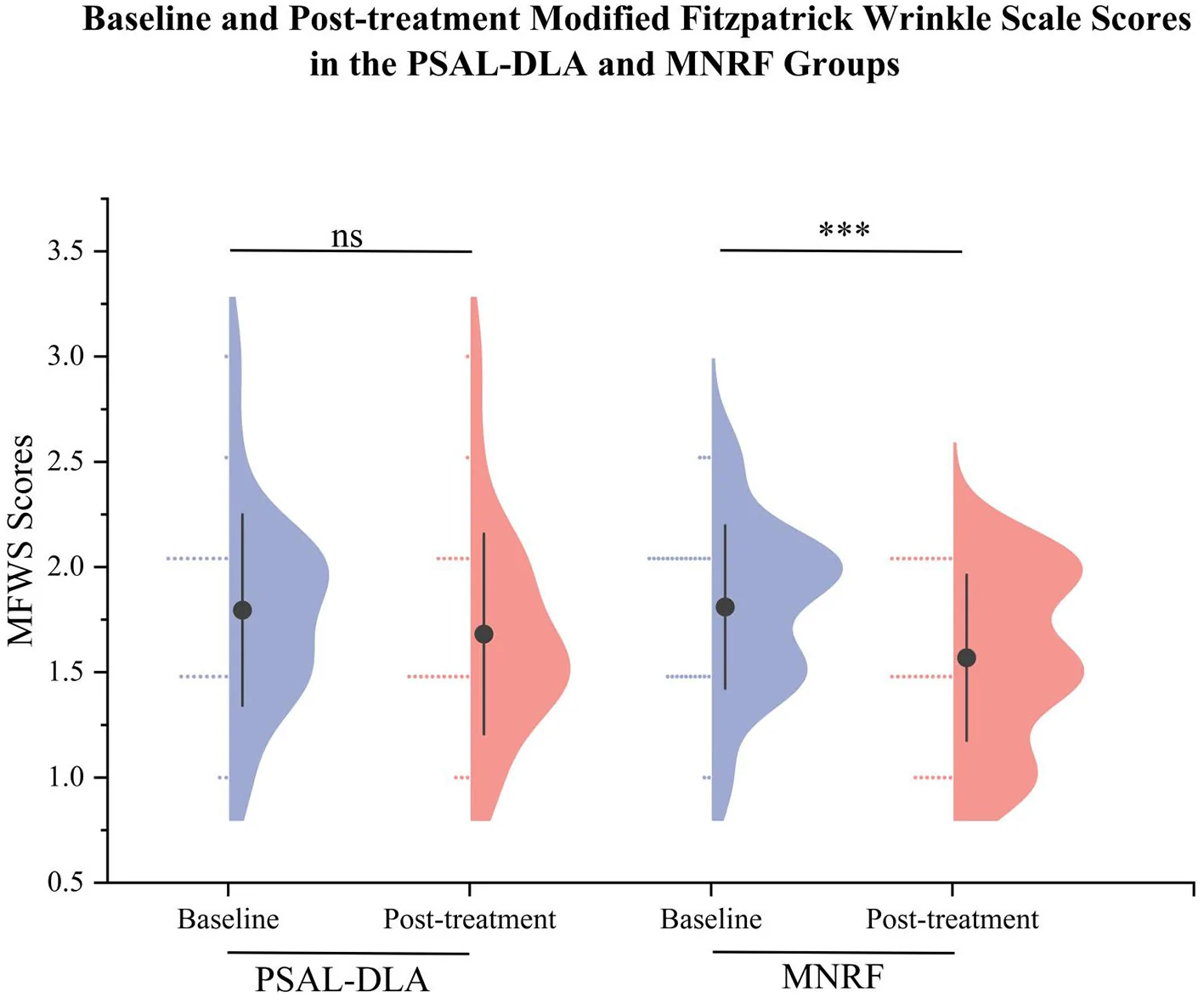
Comparisons of MFWS scores between the PSAL-DLA and MNRF groups at baseline and post-treatment. ^***^ means *p* < 0.001.

**Figure 7:**
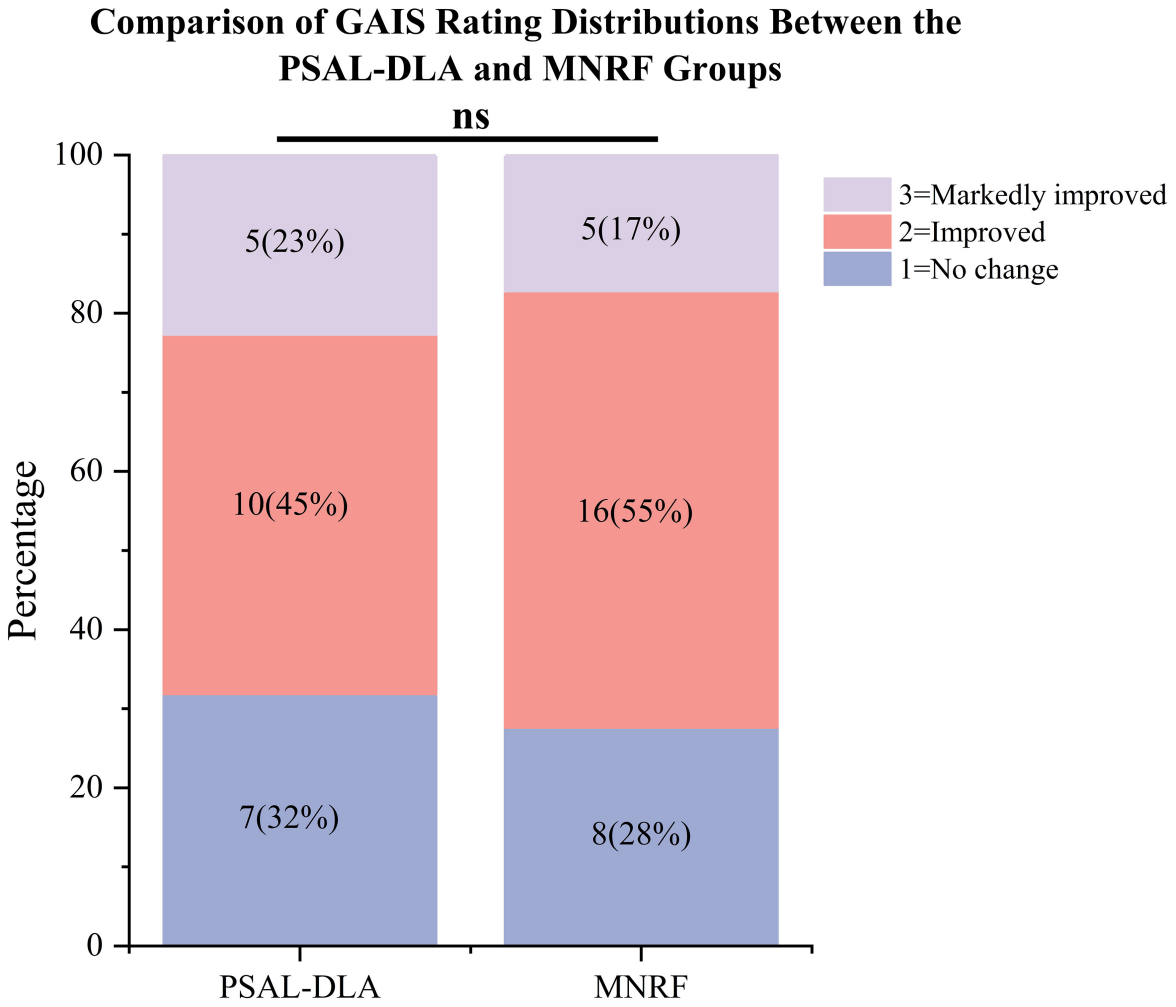
The stacked bar chart displays the percentage and number of patients in each GAIS rating.

The MNRF group showed significant improvement in MFWS score from baseline to post-treatment (Z = −3.742, *p* < 0.001), while the PSAL-DLA group did not show a statistically significant change (Z = −1.508, *P* = 0.132) (Figure 6). However, the between-group comparison of the MFWS improvement scores revealed no statistically significant difference (Z = −1.631, *P* = 0.103) (Table 5). GAIS scores showed the majority of subjects in both groups as having achieved improvement. The proportions of subjects rated as “improved” and “markedly improved” were 45.45% and 22.73% in the PSAL-DLA group, and 55.17% and 17.24% in the MNRF group, respectively (Figure 7). The comparison of overall improvement rates between the groups did not reveal a statistically significant difference (χ^2^ = 0.500, *P* = 0.779).

**Table 5:** Comparison of changes in MFWS score and GAIS scores between PSAL-DLA and MNRF.

Characteristic	PSAL-DLA (*n* = 22)	MNRF (*n* = 29)	Statistical Test	*P*-Value
**MFWS score improvements**	0.000 (-0.500, 0.000)	0.000 (-0.500, 0.000)	Z = −1.631	0.103
**GAIS scores**			X^2^ = 0.500	0.779
no change (*n*, %)	7 (31.82%)	8 (27.59%)		
Improved (*n*, %)	10 (45.45%)	16 (55.17%)		
much improved (*n*, %)	5 (22.73%)	5 (17.24%)		

### 3.3 Secondary outcomes

Subjects in both groups experienced erythema and swelling after treatment. Some subjects in the MNRF group exhibited pinpoint bleeding immediately during treatment, which ceased after brief compression. Erythema and edema were usually transient and were considered normal endpoint reactions to the treatment, resolving spontaneously in the vast majority of subjects. One subject in the PSAL-DLA group experienced persistent erythema and a burning sensation in the treated area for 5 days, which resolved after applying a topical corticosteroid as prescribed and with instructions to enhance hydration and moisturization. In the MNRF group, one subject experienced erythema and edema on the cheeks that lasted for 1 week and resolved after using a prescribed topical corticosteroid. Another subject in the MNRF group developed a 1-week acneiform eruption around the mouth, which resolved after applying a prescribed topical antibiotic. No severe long-term adverse events, such as post-inflammatory hyperpigmentation, hypopigmentation, or scarring, were observed in any subject in this study (Table 6).

**Table 6:** Summary and analysis of adverse effects.

Adverse events	Number (*n*, %)	Treatment method	Recovery time	Outcome
PSAL-DLA				
Burning or Stinging Sensations	22 (100%)	Cold Compress; Medical Dressing	2–8 h	Self-resolved
Erythema and Swelling (immediately after treatment)	22 (100%)	Cold Compress; Medical Dressing	2–24 h	Self-resolved
Erythema and Swelling (Persist in existence)	1 (4.5%)	Hormone Ointment; Medical Dressing	5 day	Resolved
MNRF				
Burning or Stinging Sensations	28 (100%)	Cold Compress; Medical Dressing	8–12 h	Self-resolved
Erythema and Swelling (immediately after treatment)	28 (100%)	Cold Compress; Medical Dressing	24–48 h	Self-resolved
Bleeding	15 (53.6%)	Brief Compression	3–5 min	Self-resolved
Erythema and Swelling (Persist in existence)	1 (3.6%)	Hormone Ointment; Medical Dressing	1 week	Resolved
Acneiform eruption	1 (3.6%)	Antibiotic Ointment	1 week	Resolved

## 4 Discussion

Facial photoaging involves UV-induced oxidative stress, collagen loss, pigment metabolism disorders, and capillary abnormalities. UVA penetrates the dermis and generates ROS, causing oxidative stress and the degradation of structural proteins, whereas UVB affects the epidermis through direct DNA damage, triggering inflammation and hyperproliferation (4). MNRF heats the deep dermis through radiofrequency energy, potentially improving wrinkles and skin texture by inducing immediate collagen fiber contraction and subsequent collagen neogenesis and remodeling (26). Previous basic research suggests that this process may involve upregulation of heat shock protein expression and downregulation of matrix metalloproteinase activity, thereby reducing collagen degradation (27, 28). Studies have also reported that epidermal thickness increases significantly after MNRF treatment, especially in the granular cell layer, which may help improve the appearance of the skin (29). However, the effect of MNRF is independent of melanin granules. While this results in a lower incidence of pigment-related adverse effects, its therapeutic efficacy on existing pigmented conditions may be suboptimal. In contrast, PSAL-DLA can precisely target and eliminate pigment particles through selective photothermolysis and LIOB, which may explain the superior improvement in pigmentation indicators such as spots and UV spots observed in the PSAL-DLA group in this study (20). The pressure waves generated by LIOB may initiate dermal collagen remodeling, and the underlying molecular mechanisms may involve the release of certain cytokines (30). Meanwhile, the 755-nm wavelength of the long-pulsed alexandrite laser can theoretically selectively target oxyhemoglobin, suggesting a potential advantage in improving telangiectasias (31). MNRF and PSAL-DLA act through two distinct mechanisms in the treatment of facial photoaging, with different efficacy emphases. It should be emphasized that this study only evaluated clinical outcomes using VISIA parameters, and the specific molecular mechanisms involved require further validation through dedicated basic research.

**Figure 8:**
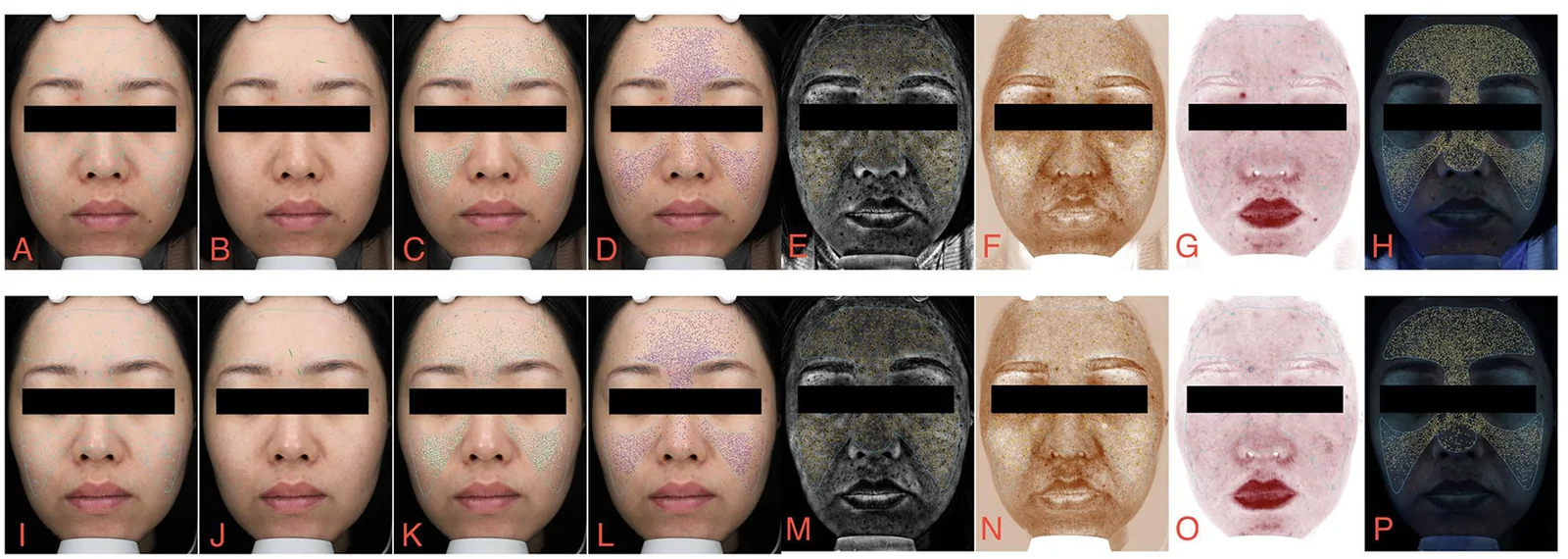
VISIA images of a 38-year-old female patient in the PSAL-DLA group. **(A-H)** Baseline VISIA images showing spots, wrinkles, texture, pores, UV spots, brown spots, red areas, porphyrins. **(I–P)** Post-treatment VISIA images showing spots, wrinkles, texture, pores, UV spots, brown spots, red areas, porphyrins.

**Figure 9:**
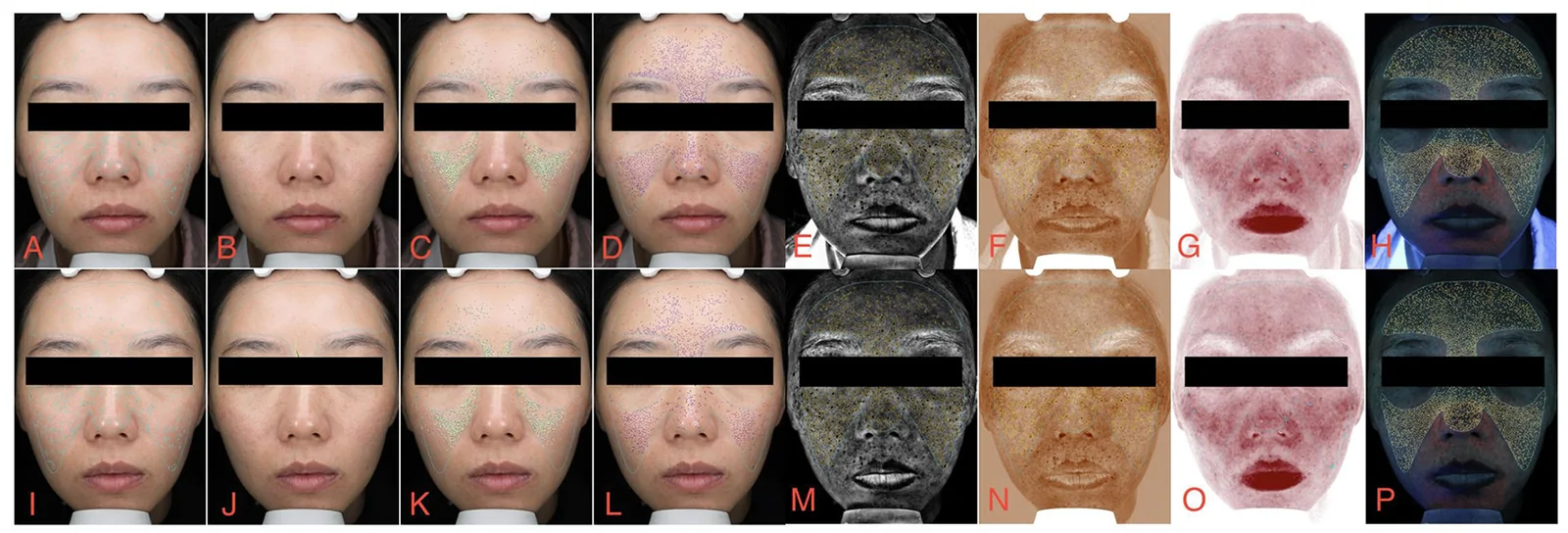
VISIA images of a 37-year-old female patient in the MNRF group. **(A–H)** Baseline VISIA images showing spots, wrinkles, texture, pores, UV spots, brown spots, red areas, porphyrins. **(I–P)** Post-treatment VISIA images showing spots, wrinkles, texture, pores, UV spots, brown spots, red areas, porphyrins.

The values of various VISIA parameters provide an objective and quantitative representation of the patient's facial skin condition. Our results showed that the PSAL-DLA group demonstrated improvements in brown spots and red areas, consistent with previous studies reporting significant reductions in pigmentation index and erythema index after treatment with PSAL-DLA combined with a long-pulsed alexandrite laser (32). At the same time, the MNRF group showed improvement in pore indicators, which is consistent with previous reports on the effectiveness of MNRF for enlarged facial pores (33). Notably, in this study, the MNRF group exhibited a statistically significant worsening of the spots score, a finding not entirely consistent with previous research, and should be considered a potential adverse outcome of MNRF treatment. The underlying mechanism may be related to post-procedural transient inflammatory responses and temporary disruption of the skin barrier. However, studies have also indicated that inflammatory cell infiltration after microneedling can persist for up to 3 months. Compared with the MNRF group, the PSAL-DLA group showed significantly greater improvement in spots and UV spots scores, highlighting its clear advantage in eliminating pigment particles and suggesting that it may be a better treatment option for pigmentary concerns. Regarding pore improvement, although a significant improvement was observed in the MNRF group in within-group comparisons, no statistically significant difference was found in the between-group comparison. This may be attributable to the inconsistent follow-up times and the relatively small sample size. The results also indicated that the degree of wrinkle improvement was positively correlated with baseline wrinkle severity in both treatment modalities, suggesting that both can effectively stimulate collagen regeneration. Furthermore, the MNRF group showed significant efficacy in the MFWS score for nasolabial fold wrinkles, consistent with its mechanism of deep heating and promotion of collagen remodeling. The GAIS scores reflect, at an overall level, that both treatments have significant efficacy for facial photoaging. Representative treatment cases are shown in Figures 8, 9.

In the PSAL-DLA group, improvements in brown spots and red areas were concentrated on the frontal view. In the MNRF group, improvements in pores were concentrated on the left and frontal

views, while the worsening of spots was concentrated on the frontal view. This may reflect differences in skin metabolism in various facial regions and variations in optical analysis from different viewing angles. Subgroup analysis showed that no significant interaction between treatment modality and age or Fitzpatrick skin type was observed in this study's sample size. However, given the limited sample size of subgroups, this result cannot rule out the possibility of clinically meaningful subgroup differences in larger samples. Additionally, as a non-invasive treatment modality for facial photoaging, picosecond laser offers the advantage of very short downtime, with most patients able to clean their face with water on the same day of treatment, reflecting its short recovery period and high selectivity.

In this study, both PSAL-DLA and MNRF demonstrated a favorable safety profile in patients, with only sporadic transient adverse reactions observed. Other studies have reported similar findings. In a group of 40 subjects treated with a 755-nm picosecond laser with a diffractive lens array, only one patient reported erythema lasting 2 days, one had edema lasting 4 days, and one developed bruising (17). Among 75 subjects treated with MNRF, three developed a rash on the forehead and five developed a rash on the cheeks, with no cases of post-inflammatory hyperpigmentation (33). The good tolerability suggests that both treatment modalities can be selected with confidence in clinical practice. At follow-up visits, it was observed that patients with a fragile skin barrier might be more sensitive to light- and energy-based treatments, and prophylactic medication post-treatment may be a good option.

This study supports the efficacy and safety of both treatment modalities, but several limitations should be considered. First, as a retrospective, non-randomized study, inherent selection bias exists, and treatment allocation may have been influenced by patients' subjective opinions, cost, and baseline photoaging condition. Second, important potential confounders, such as post-treatment skincare adherence and sun exposure, were not recorded or controlled, which could have directionally influenced pigment-related outcomes. Third, the follow-up times were inconsistent; although adjusted for in the statistical analysis, the impact of time-dependent biological processes on the results cannot be completely eliminated. Fourth, the relatively small sample size limited the statistical power for subgroup analyses. Future large-scale, prospective, randomized, split-face, double-blind, non-inferiority clinical trials with standardized treatment parameters and follow-up times are needed to further validate our findings. The current literature has not established the optimal energy density and the most appropriate number of sessions for the two modalities in treating facial photoaging. Future research can also start from this point to maximize efficacy and minimize side effects.

## 5 Conclusion

In summary, both PSAL-DLA and MNRF are effective modalities for the treatment of facial photoaging, with an overall mild adverse event profile. The two devices exhibit differentiated efficacy profiles: PSAL-DLA is more advantageous for improving pigmented indicators such as spots and UV spots, while MNRF demonstrates good efficacy for improving textural indicators such as wrinkles and pores. However, the potential transient worsening of spots after MNRF treatment should be noted and considered a potential adverse outcome. Therefore, clinical selection should not be based on the universal superiority of one device, but rather on individualized decision-making according to the patient's predominant photoaging characteristics.

## Data Availability

The data analyzed in this study is subject to the following licenses/restrictions: The raw dataset contains identifiable patient facial VISIA images and confidential clinical medical records. Public distribution is prohibited to comply with hospital data management rules and patient privacy protection requirements. Individual-level original data cannot be attached to the manuscript or shared publicly. Aggregated de-identified statistical results are only provided in the article. Data access requires formal ethical approval via the corresponding author. Requests to access these datasets should be directed to Tianhua Xu, xutian5151@163.com, No official website for data access requests, all inquiries are accepted by email with subsequent ethical review.
